# Rapid Stereochemical Analysis of Glycosylations in Flow by Ion Mobility Spectrometry

**DOI:** 10.1002/chem.202500311

**Published:** 2025-03-30

**Authors:** Jakob B. Wolf, Martin Zühlke, Dominik Weh, Marlene C. S. Dal Colle, Christian Thoben, Toralf Beitz, Klaus Bienert, Dario Cambié, Eric T. Sletten, Martina Delbianco, Stefan Zimmermann, Peter H. Seeberger

**Affiliations:** ^1^ Max Planck Institute of Colloids and Interfaces Potsdam Science Park, Am Mühlenberg 1 Potsdam 14476 Germany; ^2^ Institut für Chemie, Biochemie und Pharmazie Freie Universität Berlin Arnimallee 22 Berlin 14195 Germany; ^3^ Physical Chemistry Universität Potsdam Karl‐Liebknecht‐Straße 24–25 Potsdam 14476 Germany; ^4^ Department of Sensors and Measurement Technology Institute of Electrical Engineering and Measurement Technology Leibniz University Hannover Hannover 30167 Germany

**Keywords:** chemical glycosylation, flow chemistry, ion mobility spectrometry, reaction screening, stereoselectivity

## Abstract

Glycans are biologically important molecules that are difficult to synthesize and analyze due to their structural diversity and conformational flexibility. Stereoselective glycosylation reactions are key to achieving high‐yielding glycan syntheses. The stereochemical outcome of glycosylations is significantly influenced by factors such as the choice of activator and leaving group systems, solvent type, temperature, concentration, and stoichiometry. We introduce a flow chemistry approach to efficiently screen glycosylation conditions, using minimal material and time to enable a rapid design‐make‐test‐analyze cycle with precise parameter control for reaction optimization. Ion mobility spectrometry provides rapid separation and analysis of crude glycosylation reaction mixtures that requires less method development than liquid chromatography thus making it a valuable tool for optimizing glycosylation reactions.

## Graphical abstract

Glycans are complex molecules that are challenging to synthesize and analyze due to their diverse structures. Stereoselective glycosylation is crucial for efficient synthesis, influenced by activators, leaving groups, solvents, temperature, and concentration. A flow chemistry approach enables rapid screening and optimization, with ion mobility spectrometry (IMS) offering quick analysis of reaction mixtures. Institute and/or researcher Twitter usernames: @DelbiancoM, @mpiciPotsdam, @peterseeberger

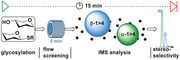

## 1 Introduction

Glycans, made up of monosaccharides linked by glycosidic bonds, can form linear or branched polymers due to the multiple hydroxyl groups on each monosaccharide. The synthesis and analysis of glycans pose a significant challenge due to their diverse structural composition and conformational flexibility.^[^ ^1^, ^2^ ^]^ Moreover, numerous types of monosaccharides differ only by the configuration of their hydroxyl groups, resulting in isomeric—and thus isobaric—forms.^[^ ^3^ ^]^

High‐yielding chemical synthesis of oligosaccharides and polysaccharides depends on achieving high stereoselectivity during glycoside formation. Stereoselective formation of 1,2‐*trans* glycosidic linkages benefits from the use of neighboring group participation (NGP). In contrast, 1,2‐*cis* glycosylation requires meticulous optimization to achieve high stereoselectivity.^[^ ^4^, ^5^ ^]^ Yield and stereoselectivity are affected by multiple parameters, such as activator and leaving group, solvents,^[^ ^6^ ^]^ temperature,^[^ ^7^, ^8^ ^]^ concentration, and stoichiometry.^[^ ^9^ ^]^ Screening these parameters can be complicated by thermal gradients at low temperatures,^[^ ^10^ ^]^ operator errors, and required operator time. Subsequent reaction analysis often requires extensive sample preparation, slowing down the optimization process and influencing yield or anomeric ratios, especially in the case of preparative column isolation.

**Figure 1:**
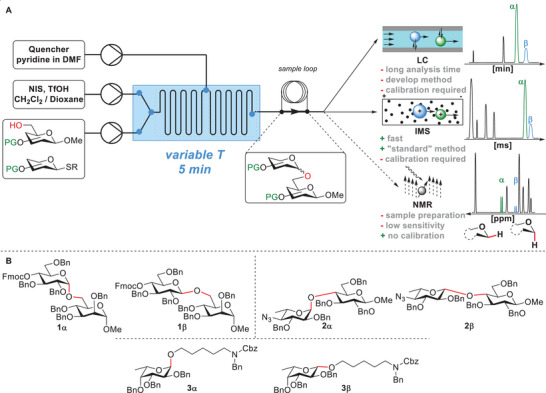
(A) Schematic view of the flow platform and comparison of different analytical methods. (B) Stereoisomers of the investigated target compounds.

To address these challenges and streamline the optimization of glycosylations, a well‐defined reaction procedure with precise parameter control, ideally within a rapid design‐make‐test‐analyze (DMTA) cycle would be beneficial. The data should be accurate and reproducible, with minimal human interaction and minimal need for extensive analytical method development. Such an approach would simplify glycosylation optimization and allow for a better understanding of various effects by higher throughput and reduced variation, ultimately improving the installation of glycosidic linkages with desired stereochemical configurations. Here, we present a flow chemistry approach to facilitate time‐ and resource efficient screening of the parameters for glycosylation reactions within less than 20 min while requiring only a small amount of material (<7 mg of glycosyl donor for each experiment). For this screening approach, we compare analytical methods in terms of sample amounts required, analysis time, if directly interfaceable with the flow system, and comparability of results (Figure 1).

Combining rapid reaction and rapid analysis results in a good DMTA cycle. Continuous parameter change and monitoring of the reaction outcome with sufficiently fast analytical method can allow for construction of dynamic reaction trajectories, thereby saving time and material.^[^ ^11^ ^]^ A rapid technique for continuous monitoring of anomeric ratios of glycosides would be desirable.

Optimization in chemical glycosylations commonly employs NMR and LC‐MS for glycan analysis. The glycoside products typically differ only in one stereocenter and often require extensive separation times for liquid chromatography (LC), complicate the interpretation of nuclear magnetic resonance (NMR) data, and limit the usability of mass spectrometry (MS) due to the presence of isobaric anomers that cannot be separated based on their mass‐to‐charge ratio. With an internal standard, NMR can directly provide quantitative results,^[^ ^12^ ^]^ but the sensitivity of routine NMR analysis requires a significant quantity of material and low spectral dispersion in glycans can hamper quantification.^[^ ^2^ ^]^ The presence of solvent, unreacted starting materials, reagents, or by‐ and side‐products can result in peak overlap in crude mixtures, frequently necessitating workup or column purification. Thus, online monitoring of small‐scale glycosylations by NMR is complicated. Commonly, LC‐based analysis requires less material than NMR, but is time‐consuming, particularly for anomeric separation, and not universally applicable: Different glycosylation products often necessitate specific conditions for anomeric resolution, increasing complexity, analysis and development time. Each product typically requires separate calibration, and significant amounts of high‐purity solvents are required, making LC time‐consuming, costly and wasteful, particularly in large‐scale screening applications.

Ion mobility spectrometry (IMS) offers rapid separation based on collisional‐cross‐section‐to‐charge ratio and for glycan analysis is often combined with MS to separate by mass‐to‐charge ratio.^[^ ^13^, ^14^ ^]^ Despite relatively poor orthogonality of the separation principles,^[^ ^15^ ^]^ different drift times in the IMS offer additional separation of the same MS ion mass. IMS‐MS thereby allowed for stereo‐ and regiospecific analysis of underivatized glycans^[^ ^16^, ^17^, ^18^, ^19^ ^]^ and separation of isomeric oligosaccharides.^[^ ^20^, ^21^, ^22^, ^23^, ^24^ ^]^ Metal adducts enabled the separation of oligosaccharide isomers,^[^ ^21^, ^25^ ^]^ and even diastereomeric and enantiomeric monosaccharides were resolved as peptide or amino acid adducts.^[^ ^26^ ^]^ Under atmospheric pressure conditions, different di‐ and trisaccharides were separated,^[^ ^27^ ^]^ to replace expensive IMS‐MS by high‐resolution stand‐alone or multistage IMS operating.^[^ ^28^, ^29^ ^]^ Smaller, mobile stand‐alone devices do not require large vacuum pumps. LC‐IMS is comparable to LC‐LC, but can reduce separation times for complex mixtures by a factor of ten.^[^ ^30^, ^31^, ^32^ ^]^ Until now, IMS has been exclusively used to analyze methyl glycosides,^[^ ^33^, ^34^ ^]^ partially acetylated glycans,^[^ ^35^, ^36^ ^]^ and fully protected glycan mono‐ and disaccharides.^[^ ^37^ ^]^ Despite advances in glycan analysis by IMS, direct analyses of crude reaction mixtures have not been reported.

To determine whether IMS can be used to assess the stereoselectivity of glycosylation reaction mixtures, LC‐ELSD/MS analysis, offline ^1^H‐NMR and at‐line IMS were compared. The glycosylation reactions are conducted in a chip flow reactor that offers precise temperature control via an attached recirculating chiller and reduced variability, as reagent addition and stoichiometry are precisely controlled by flow rates.^[^ ^38^ ^]^ Reaction conversion and selectivity were derived from LC–ELSD peak areas identified via MS analysis. We show that IMS analysis can provide a faster, workup‐free assessment of stereoselectivity without the need of extensive method development with a cost‐effective, small, mobile, and robust device, enabling a fast DMTA cycle.

## 2 Results and Discussion

Three glycosylation reactions were chosen as models to compare IMS to established analytical techniques (Figure 1B). Two disaccharides (**1α/1β** and **2α/2β**) as well as the fucosylation of an alkanolamine linker (Figure 1, **3α**/**3β**) that presents analytical challenges due to the high flexibility of the alkyl portion were investigated. Most glycosylations were performed in flow, with some reactions performed in batch to study discrete variables such as solvents or to test transferability of results. The flow reactions were performed in a custom‐built system with a temperature‐controlled glass chip reactor and three solution inputs (Figure 1A). Once the reactor reached its target temperature, three syringe pumps delivered the solutions at a flow‐rate providing a 5 min residence time. Syringe pump 1 delivered an equimolar combination of thioglycoside donor and a hydroxyl‐bearing acceptor in CH_2_Cl_2_. To initiate the reaction, the reagent solution was mixed with an activator solution composed of *N*‐iodosuccinimide (NIS)/trifluoromethanesulfonic acid (TfOH) in CH_2_Cl_2_ with 5 vol% 1,4‐dioxane to achieve homogeneity delivered by syringe pump 2.^[^ ^39^, ^40^, ^41^, ^42^ ^]^ The reaction was then quenched by a solution of pyridine (1.2 vol%) in dimethylformamide (DMF) from the third syringe before entering the analytical section. The reaction was sampled with an injection valve after 2.5 residence times to ensure steady state and analyzed via LC‐ELSD (column: C18, mobile phase: MeOH/water with 0.1% formic acid, 15 min, see Supporting Information for further details). The reactor effluent was collected for an additional 10 min into a vial and reanalyzed offline with LC‐ELSD, and LC‐UV‐Vis, complemented by NMR and IMS. An aliquot of the collected reactor effluent was concentrated under reduced pressure, the residue dissolved in CDCl_3_, and submitted for NMR analysis. For the IMS measurements, the reactor effluent was diluted with a solution of water in acetonitrile (20 vol%) to a concentration of 1–10 vol%. The diluted sample was manually filled into a sample loop and injected into a home‐built IMS operating at a system temperature of 150 °C (154 mm drift length at a field strength of 62 V/mm) with a constant flow of solvent (5 µl/min).^[^ ^43^ ^]^

To investigate whether the conditions and results found in flow are reproducible in batch, and to check discrete reaction parameters, batch reactions were performed in an ice bath (−18 °C, ice/NaCl) in sealed glass vials. Donor and acceptor were added as an equimolar solution in one of the following solvents: CH_2_Cl_2_, MeCN, or MTBE. After equilibrating the temperature for 5 min, the activator was slowly added via a syringe. The reactions were quenched after 5 min and treated in the same way as the outlet of the flow reactor and then analyzed.

### 2.1 Model Glycosylation **1**

**Figure 2:**
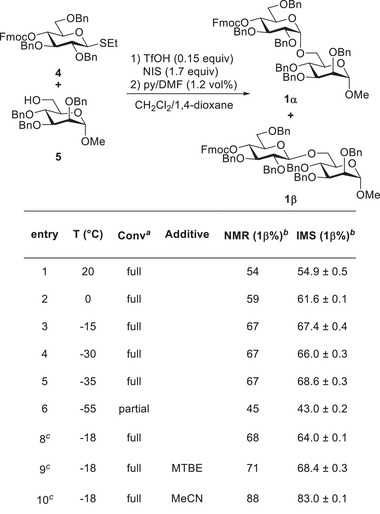
1,6‐Glycosylation of mannoside **5** with thioglycoside **4** afforded different ratios of anomers **1α** and **1β**. Depending on reaction temperature and solvent mixture, the reaction time was fixed at 5 min. IMS ratios are from volume integration and threefold measurement of each sample, with the error in percent (standard deviation/mean value). ^[a]^Conversion is based on disappearance of the starting material in LC‐ELSD. ^[b]^Anomeric ratio is expressed as 100% × A(**1β**)/(A(**1α**) + A(**1β**)). ^[c]^Reactions in different solvents were performed in batch due to ease of screening discrete variables (see Supporting Information for further information).

The glycosylation of 6‐OH mannoside **5** with donor **4** was evaluated, and complete conversion was achieved within 5 min, with a slight increase in β‐selectivity at lower temperatures (Figure 2, entries 1–5). A reduction in the temperature to −55 °C (Figure 2, entry 6) resulted in incomplete conversion and diminished β‐selectivity. In all trials, the stereoselectivity analyses by NMR and IMS showed good agreement. While LC failed to easily resolve the anomers, IMS achieved anomer separation within 1 min including system flushing and injection. The glycosylation was also performed in batch conditions to compare different solvents in sealed vials at −18 °C. Baseline experiments in CH_2_Cl_2_ (Figure 2, entry 8) were compared to glycosylations in MTBE (Figure 2, entry 9) and MeCN (Figure 2, entry 10). The baseline experiment illustrated that batch (Figure 2, entry 8) and flow experiments (Figure 2, entry 3) are comparable and no significant change was observed with MTBE (Figure 2, entry 9). The addition of MeCN in the batch process led to a significant change in stereoselectivity, providing the highest β‐selectivity observed (Figure 2, entry 10).^[^ ^44^ ^]^ Conversely, the lowest β‐selectivity was attained by conducting the reaction at −55 °C in flow (Figure 2, entry 6). This exploration underscores the nuanced impact of temperature and solvent choice on the efficiency and selectivity of the glycosylation process.^[^ ^7^, ^8^, ^9^, ^10^, ^45^, ^46^ ^]^ Furthermore, it shows IMS analysis of an aliquot of quenched crude material obtained from batch‐based screening by simply diluting. This highlights wide applicability in glycochemistry, where the prevalent method is batch‐based synthesis.

### 2.2 Model Glycosylation **2**

**Figure 3:**
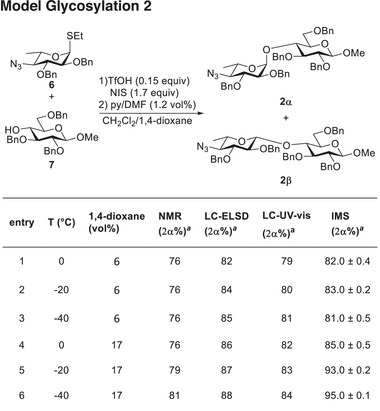
Glycosylation of **7** with azido‐protected building block **6** in flow. Full conversion by disappearance of thioglycoside was observed in all cases after 5 min residence time. Anomeric ratios at different temperatures and with different concentrations of 1,4‐dioxane. IMS ratios are from volume integration and threefold measurement of each sample, with the error in percent (standard deviation/mean value). ^[a]^Anomeric ratios are expressed as 100 × A(**2α**)/(A(**2α**) + A(**2β**)).

The stereochemical outcome of the glycosylation involving monosaccharides **6** and **7** was found to be independent of the reaction temperature (Figure 3, entries 1–3). All analytical techniques showed similar results in good agreement with previous batch experiments (see Supporting Information, Table S3). Three experiments were conducted in flow at different temperatures to study the effect of higher dioxane concentration (Figure 3, entries 4–6). In this case, all analytical techniques were in good agreement, showing a slight temperature dependence of the glycosylation outcome, with some minor fluctuation to slightly higher selectivity in IMS with decreasing temperature (Figure 3, entries 4–6). Since homogenous reaction conditions are required for flow based screenings,^[^ ^47^ ^]^ some 1,4‐dioxane is required in all reactions, this changes the parameter space from binary to continuous.

### 2.3 Limitations

**Figure 4:**
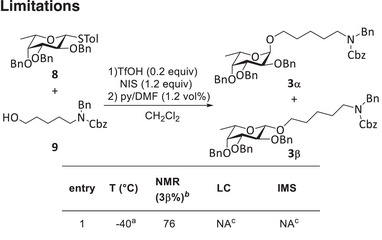
Batch glycosylation of protected 1‐aminopentan‐5‐ol **9** with fucose building block **8** in batch. ^[a]^The reaction was performed in a cooling bath. ^[b]^Anomeric ratios are expressed as 100*A(**3α**)/(A(**3α**)+A(**3β**)). ^[c]^Anomers failed to separate as a mixture of anomers afforded one peak.

The analysis of crude glycosylation of protected aminopentanol linker affording anomers **3α** and **3β** (Figure 4) resulted in indistinguishable drift times in IMS and afforded a single peak. Likely the low difference in CCS is caused by the highly flexible alkylamine chain. Calculation of the CCS could potentially predict separability by IMS.^[^ ^48^ ^]^ In this case, IMS cannot speed up the DMTA cycle because anomers do not separate. Since separation of **3α** and **3β** on a C18 column using a standard method also failed, the anomeric ratio could only be derived from manual workup and ^1^H‐NMR. No further studies on temperature and solvent effects were conducted on this target molecule.

### 2.4 Analytical Technique Comparison

NMR quantification was straightforward using routine 1H‐NMR equipment as calibration is not necessary and even quantitative assessment of yield is possible when an internal standard is used. For disaccharides **1α/1β** and **2α/2β**, spectral congestion and peak overlap hampered direct signal integration, requiring peak deconvolution to determine the anomeric ratio. NMR quantification was possible in all cases studied, but required solvent removal and comparatively high sample amounts (5 µmol were used in study, see Supporting Information for respective calculation). This method is comparatively slow and material consuming, since the sample needs to be collected, worked up, measured and analyzed manually.

LC derived ratios can assist in anomeric ratio estimation. Our method was based on reverse‐phase C18 core shell column with water/methanol gradient elution within 15 min. This method sufficed to separate compounds **2α** and **2β**, however, failed to separate anomeric mixtures of **1α/1β** and **3α/3β**. Many other stationary and mobile phases exist and anomeric resolution may be achieved for a given protected glycan after careful selection and tuning, but method development is required each time. While little material was sufficient for analysis (5 nmol of sample were used in this study, see Supporting Information for respective calculation) quantitative determination of reaction yields is only possible after calibration. LC is more easily integrated into flow platforms than NMR and suitable for a fast DMTA cycle.

IMS analysis required little material (2.5 nmol sample, see Supporting Information for respective calculation) and gave fasts results (1 s, see Supporting Information for respective calculation) after calibration. The method performed well for all the disaccharides, but failed to achieve anomeric resolution of compounds **3α** and **3β**, containing a flexible aglycon, did not resolve with our LC method. Integration of peak volumes over time provided lower standard deviation and better agreement with other studied analytical methods. This may be due to small time delays between intensity maxima of ion species at the IMS detector. Albeit that we performed only at‐line IMS in this study, IMS is expected to be relatively easily integrated into flow platforms, requiring a similar interface as LC, an injection valve and a make‐up/dilution pump and could, where applicable, support a fast DMTA cycle.

## 3 Conclusion

IMS was employed for the analysis of crude glycosylation mixtures of fully protected glycans. The results were in good agreement with other analytical methods. The IMS analysis required no method redevelopment, less time than LC and simpler sample workup and lower sample quantities than NMR. The combination of LC and IMS will allow for rapid separation of anomers and assist in rapid reaction optimization. Hyphenation to LC may reduce ionization competition and enhance accuracy. Comparability between online and offline analytical results ensured steady‐state conditions and suitability for larger scale preparation of target compounds by prolonged flow experiments. *De novo* optimization could use computationally derived CCS,^[^ ^49^, ^50^ ^]^ however, complications in this approach could arise from charge carrier size and ionization site influencing CCS.^[^ ^22^ ^]^ Expanding existing databases like GlycoMob^[^ ^51^ ^]^ will help with the assignment of product and side‐products. Despite facing challenges related to instrument sensitivity, IMS can speed up optimization of stereoselectivity in glycosylations and potentially enable closed‐loop optimization.

## Supporting Information

The authors have cited additional references within the Supporting Information.^[52–67]^

## Author Contributions

Jakob B. Wolf conducted the experiments, wrote the manuscript. Eric T. Sletten, Jakob B. Wolf, Dario Cambié, Martin Zühlke devised and managed the project. Martin Zühlke conducted and analyzed IMS experiments. Toralf Beitz and Stefan Zimmermann helped with interpretation of IMS results. Christian Thoben and Stefan Zimmermann developed and supplied the IMS spectrometer. Klaus Bienert developed the Peltier cooling unit. Peter H. Seeberger supervised the project, edited the manuscript and secured funding. Martina Delbianco, Marlene C. S. Dal Colle, and Dominik Weh provided material and helped preparing the Supporting Information.

## Conflict of Interests Statement

The authors declare no conflict of interest.

## Supporting information



Supporting Information

## Data Availability

The data that support the findings of this study are available in the supplementary material of this article.
