# Supplementary material for: Rapid Stereochemical Analysis of Glycosylations in Flow by Ion Mobility Spectrometry
Source: Chemistry. 2025 Mar 30;31(25):e202500311. doi: 10.1002/chem.202500311 (PMC12057612; doi:10.1002/chem.202500311)
Supplement: Supplementary file 1 — Supporting Information [file CHEM-31-e202500311-s001.docx]

Rapid Stereochemical Analysis of Glycosylations in Flow by Ion Mobility Spectrometry

Jakob B. Wolf,^[a], [b]^ Martin Zühlke,^[c]^ Dominik Weh,^[a], [b]^ Marlene Dal Colle,^[a], [b]^ Christian Thoben,^[d]^ Toralf Beitz,^[c]^ Klaus Bienert^[a]^, Dario Cambié,^[a]^ Eric T. Sletten,^[a]^ Martina Delbianco,^[a]^ Stefan Zimmermann,*^[d]^ Peter H. Seeberger^[a]^*

[a] M. Sc. Jakob B. Wolf, M. Sc. Dominik Weh, M. Sc. Marlene Dal Colle, Klaus Bienert, Dr. Dario Cambié, Dr. Eric T. Sletten, Dr. Martina Delbianco, Prof. Dr. Peter H. Seeberger

**Max Planck Institute of Colloids and Interfaces**

**Potsdam Science Park**

**Am Mühlenberg 1
14476 Potsdam**
E-mail: peter.seeberger@mpikg.mpg.de

[b] M. Sc. Jakob B. Wolf, M. Sc. Dominik Weh, M. Sc. Marlene Dal Colle
Institut für Chemie, Biochemie und Pharmazie
Freie Universität Berlin
Takusstraße 3

14195 Berlin

[c] Dr. Martin Zühlke, Dr. Toralf Beitz
Physical Chemistry

Universität Potsdam

Karl-Liebknecht-Straße 24-25

14476 Potsdam

[d] M. Sc. Christian Thoben, Prof. Dr.-Ing. Stefan Zimmermann
Department of Sensors and Measurement Technology,

Institute of Electrical Engineering and Measurement Technology

Leibniz University Hannover
30167 Hannover

Germany

Contents

[Experimental 3](#_Toc187829651)

[General Considerations 3](#_Toc187829652)

[Chemicals 3](#_Toc187829653)

[Analytical and spectroscopic procedures 3](#_Toc187829654)

[IMS conditions 4](#_Toc187829655)

[Experimental Procedure 5](#_Toc187829656)

[Setup 5](#_Toc187829657)

[Fluidic Connections 6](#_Toc187829658)

[Reactant delivery 6](#_Toc187829659)

[Glycosyl donor, acceptor and activator preparation 6](#_Toc187829660)

[Experiment Execution 7](#_Toc187829661)

[Syntheses 8](#_Toc187829662)

[Synthesis of donor 6 (ethyl 2,3-*O*-dibenzyl-4-azido-6-deoxy-thio-α/β-L-glucopyranoside) 8](#_Toc187829663)

[Synthesis of analytical reference for disaccharide 2 (2,3-*O*-dibenzyl-4-azido-6-deoxy-β-L-glucopyranosyl-(1→4)2-*O*-methoxy-3,4,6-*O*-tribenzyl-ß-D-glucopyranoside): 27](#_Toc187829664)

[Synthesis of analytical reference for compound 3 34](#_Toc187829665)

[Synthesis of analytical reference of disaccharide 1 (2,3,6-O-Tribenzyl-4-O-Fluorenylmethoxycarbonyl-D-glucopyranosyl-(1→6)-O-1-methoxy-2,3,4-O-tribenzyl-a-D-mannopyranoside) 41](#_Toc187829666)

[Raw data of Analysis of Glycosylations 48](#_Toc187829667)

[Model Glycosylation 1 at different temperatures 48](#_Toc187829668)

[Model Glycosylation 1 with different solvent additives 56](#_Toc187829669)

[Model glycosylation 2 at different temperatures 60](#_Toc187829670)

[Model Glycosylation 3 (crude reaction mixture ^1^H NMR) 73](#_Toc187829671)

[References 74](#_Toc187829672)

# Experimental

## General Considerations

All air- and water-sensitive reactions were performed under argon atmosphere and using solvents dried according to literature and their water content verified to be <5 ppm by Karl-Fischer titration.^[52]^ Molecular sieves 3 Å were activated at 180 °C under reduced pressure overnight and stored under inert gas. The reaction progress was monitored by TLC carried out on silica gel 60 F254 plates (Merck, Darmstadt) and visualized by UV-detection at 254 nm and 360 nm or **dipping the plate in one of the following stains: cerium molybdate stain (CAM, 0.5 g of Ce(NH_4_)_4_(SO_4_)_4_·H_2_O, 12 g (NH_4_)_6_Mo_7_O_24_, and 15 mL H_2_SO_4_ in 235 mL H_2_O) or H_2_SO_4_ stain (H_2_SO_4_ 10%m in EtOH). Flash column chromatography was carried out using forced flow of eluent on silica gel (Macherey-Nagel Silica 60, 0.04 - 0.63 mm).**

## Chemicals

**All chemicals used were reagent grade and used as supplied unless otherwise noted. The following compounds were synthesized adapting literature procedures: S2**^[53]^, **S3**^[54,55]^, **S4**^[54]^, **S5**^[56,57]^, **6**^[57]^. Compounds **4**^[61]^, **5**^[60]^, **7**^[58]^, **9**^[59]^ were prepared according to literature procedures and matched reported characterization**.** Glycosyl donor **8** was purchased from GlycoUniverse (Germany, product code LFuc32.020202). Solution phase glycosylation of glycosyl acceptor **7**^[58]^ was conducted with glycosyl donor **6**. Solution phase glycosylation of acceptor **9**^[59]^ was conducted with glycosyl donor **8**. Solution phase glycosylation of acceptor **5**^[60]^ was conducted with glycosyl donor **4**^[61]^. *N*-Iodosuccinimide was recrystallized from hot 1,4-dioxane and stored in the fridge. Peptide synthesis grade DMF was used.

## Analytical and spectroscopic procedures

**^1^H, ^13^C, HSQC and COSY spectra were recorded on an Ascend^TM^ 400 spectrometer (400 MHz, cryoprobe, Bruker) or Varian^TM^ 400 (400 MHz, Agilent). Chemical shifts (*δ*) are reported in parts per million (ppm). Spectra were recorded using the residual proton solvent peak as reference in CDCl_3_ (CHCl_3_: 7.26 ppm ^1^H, 77.16 ppm ^13^C) or D_2_O (H_2_O: 4.79 ppm ^1^H). The following abbreviations are used to indicate peak multiplicities:** s singlet; br s broad singlet; d doublet; dd doublet of doublets; t triplet; dt doublet of triplets; td triplet of doublets; q quartet, p pentet, m multiplet. Coupling constants (*J*) are reported in Hertz (Hz). All mixture of anomers are expressed in percent of major anomer of total target compound: Anomer_Major_ [%] = 100% * Anomer_Major_ /( Anomer_Major_ + Anomer_Minor_). NMR spectra were evaluated using MestReNova 14.3.0-30573 (MestReLab Resarch S.L). **Assignments in ^1^H and ^13^C NMR spectra were done using HSQC and COSY NMR spectra. Mass spectrometry was performed on Agilent 1260 Infinity II series. High resolution mass spectrometry by electrospray ionization (ESI-HRMS) was carried out using a Xevo G2-XS QTof (Waters) using positive ion mode. Time-of-flight by matrix assisted laser desorption (MALDI-TOF) was performed on Autoflex^TM^ (Bruker) using positive ion mode. Samples for MALDI-TOF were prepared using dihydroxybenzoic acid (DHB) solution (20 mg DHB in 1 mL of water) as a matrix (2 µL) mixed with a solution of the sample (2 µL). Liquid chromatography was performed using LC-MS grade solvent and modifiers and ultra-pure water obtained from an in-house system (Milli-Q^®^, Millipore Corporation). Online and offline** liquid chromatography was performed on a Knauer PlatinBlue (Knauer GmbH, Berlin, Germany) LC system, using a C18 Core-shell column (C18 meteoric core, 2.1 mm I.D.* 100 mm, 8 nm S 2.7 µm, YMC Co. Ltd.) and a SEDEX-LC LT-ELSD (Sedere, France) as detector set to 50 °C and measuring at a frequency of 30 Hz. A splitter (Micro-Splitter Valve Assembly, Idex) was used to divert the flow between the ELSD (475 µL/min) and the MS (25 µL/min). A Xevo-TQ MS (Waters, USA) was used for mass detection. Analysis was performed using gradient elution over 15 min (see Table 1 for details) with mobile phase composed of water and methanol both with 0.1 v% formic acid as modifier. Offline LC-UVVis/MS analysis was performed on a Waters Acquity UPLC system with UV detector (TUV, Waters) and Xevo TQ-S (Waters) as mass detector. A C18 column (Acquity UPLC BEH C18 1.7 µm, 2.1 mm x 100 mm, Part No. 186002352) was used as stationary phase and acetonitrile and water both with 0.1 v% formic acid as modifier provided a gradient over 12 min for separation.

Table 1: Details on gradients used on the two LC systems

| LC- ELSD gradient @ 0.5 mL/min | | LC-UVVis gradient @ 0.2 mL/min | |
| --- | --- | --- | --- |
| Time [min] | methanol [%] | Time [min] | acetonitrile [%] |
| 0 | 50 | 0 | 1 |
| 1 | 50 | 5 | 95 |
| 7 | 90 | 10 | 95 |
| 10 | 100 | 11 | 1 |
| 13 | 100 | 12 | 1 |
| 14 | 50 | - | - |
| 15 | 50 | - | - |

## IMS conditions

A home-built ion mobility spectrometer^[62]^ with an increased system temperature of 150 °C and an extended drift length of 154 mm at a drift field strength of 62 V/mm was utilized. The solvent was introduced into the system via a syringe pump (KDS-200-CE, KD Scientific Inc., Holliston, Massachusetts, USA) at a constant flow rate of typically 5 μL/min. The syringe outlet is connected to a six-port two-position injection valve with a 5 μL sample loop, in injection position the solvent flow feeds the sample into the capillary of the heated electrospray ionization (HESI) source (gauge 34, OPTON-20037, Thermo Fisher Scientific, Dreieich, Germany). Coaxial sheath gas from 0 to 3 L/min and heating gas from 0 to 1 L/min with gas temperatures between 20 °C and 300 °C could be applied. Nitrogen N5.0 (Nippon Gases Deutschland GmbH, Berlin, Germany) is employed as both sheath and heating gas. Sample was prepared by diluting crude reactor outlet (100 µL) with a water:acetonitrile (2:8) mixture (900 µL) resulting in a 10 v% solution of sample and the sample loop was filled via a syringe (Hamilton, 10 ml, Model 701 N, 26 s gauge, Bonaduz, Switzerland) manually. In case lower concentration was necessary, thus obtained 10% sample solution was further diluted to 1% following the same procedure.

IMS Area vs volume integration

Glycosylation **2** was evaluated by IMS analysis, a comparison between volume-based integration and area-based integration is provided in Table S1.

Table S2: IMS area (2D) or volume (3D) of 2α in glycosylation of 2. Area/volume were determined after baseline correction and normalization to total area/volume of 2α and 2β: 2α/(2α+2β). Mean and relative standard deviation over three respective four (marked with *) repetitions of IMS measurement of crude reaction solution afforded from different reaction conditions.

| Conditions | 2D | | 3D | |
| --- | --- | --- | --- | --- |
|  | Mean | RSD [%] | Mean | RSD [%] |
| 0 °C, 6 v% 1,4-dioxane | 0.9473 | 1.5 | 0.8181 | 2.6 |
| −20 °C, 6 v% 1,4-dioxane* | 0.8895 | 3.4 | 0.8336 | 1.4 |
| −40 °C, 6 v% 1,4-dioxane* | 0.7576 | 7.1 | 0.8139 | 3.1 |
| 0 °C, 17 v% 1,4-dioxane | 0.8716 | 3.8 | 0.8451 | 3.2 |
| −20 °C, 17 v% 1,4-dioxane | 0.7399 | 1.1 | 0.9261 | 1.9 |
| −40 °C, 17 v% 1,4-dioxane | 0.7060 | 1.1 | 0.9527 | 0.4 |

In the specific case of IMS analysis, the occurrence of a double peak for **2β** suggests the presence of protomers^[63]^ or reflect a differential affinity to sodium ions.^[64,65]^ Such observations underscore the complexity of molecular interactions in analytical chemistry and highlight the need for further investigation.

## Experimental Procedure

### Setup


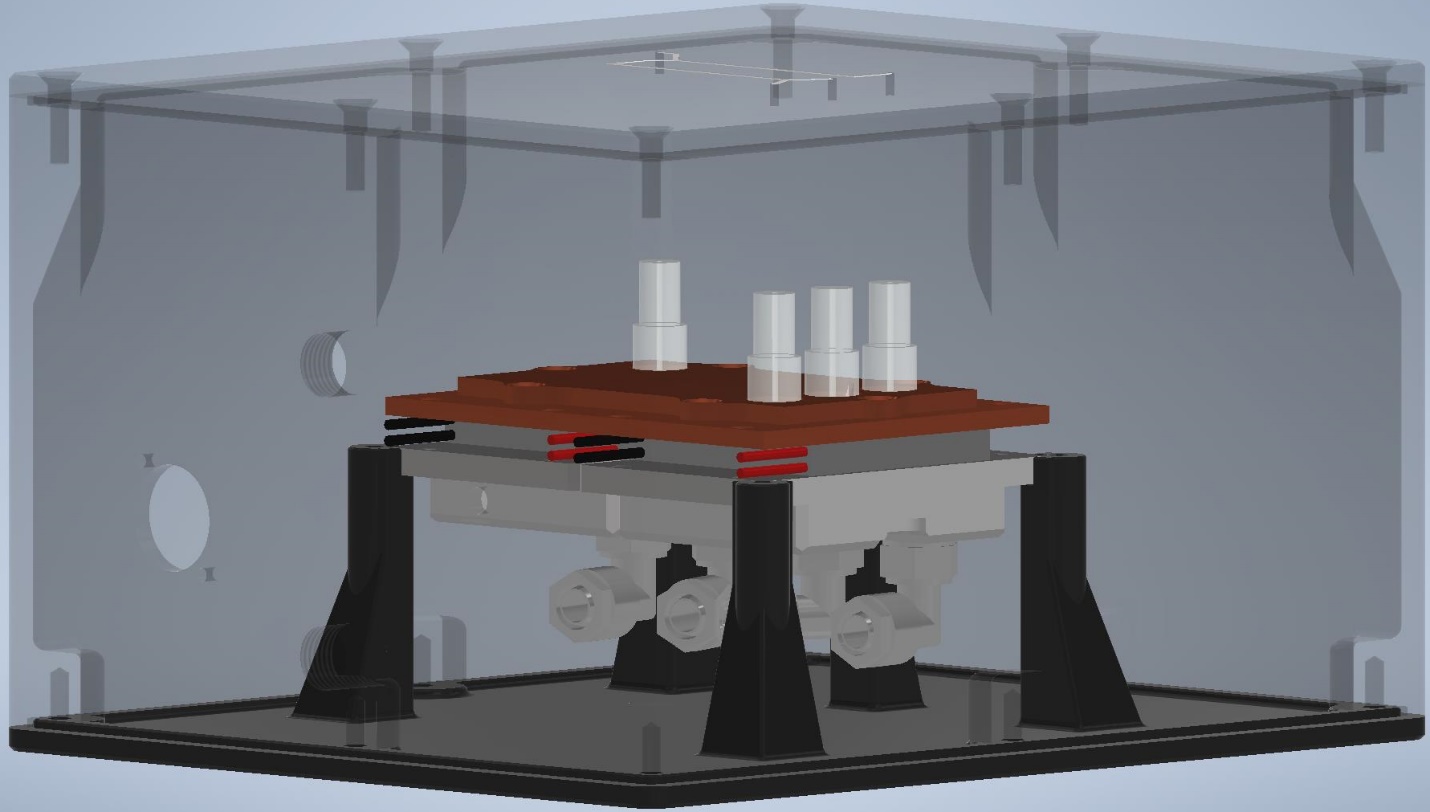


Figure S1: Reactor sandwiched between two copper plates with four peltier elements in two pairs providing temperature control. Excess heat of peltier elements is removed by heatsinks connected to a cooling fluid stream, on the bottom. The whole setup is put inside of an insulating box filled with aerogel.

The flow glycosylation was performed in a glass chip microreactor (Micromixer * Type ST-3-1 components, Little Things Factory GmbH, Elsoff, Germany) with an internal volume of 58 µL for the reaction compartment and 46 µL for the quenching compartment. The glass reactor was sandwiched between two copper plates, the top one was equipped with a PT-1000 probe pressed onto the glass reactor.

The reactor temperature was controlled via a Peltier cooler built in-house (see Figure S1). Four Peltier elements (order code UEPT-555-241-060D200, I_max_ = 6 A, U_max_ = 34 V, T_max_ = 200 °C, dT_max_: 78 K, Uwe electronic GmbH) were sandwiched in pairs, the upper pair (facing towards the reactor) was connected in series, the two bottom ones (connected to the cooling plate) connected in parallel. Two heat sinks (X-Flow XEON, Innovatek OS GmbH, Germany) were cooled by a recirculation chiller (RC-10 Digital Chiller, VWR) set to −10 °C. Temperature was controlled at constant potential (24 V) by varying the current (controller: TEC16-24, Head Electronic, power supply: PPL300U-240 with 24 V/DC, 12.50 A, 300 W, Phihong). The target temperature was measured on top of the reactor with a PT-1000 probe (VARIOHM ERTD2-PT-1000-A-3850, Variohm Eurosensor Ltd), and as a safety measure, another PT-1000 probe of the same type was installed in the heatsink to switch the power supply off in case of over temperature. The experiment was started once the measured temperature measured on top of the reactor was within 1 °C of target value. The reactor and Peltier cooler was kept inside a 3D-printed box filled with aerogel (Kwark^®^ GL, Enersens, France) as thermal insulation material. Construction files are available from the authors upon request. To analyze the reaction solution, a sample loop (1 µL) is connected to the LC system with a six port two-position injection valve (Cheminert C72H-1696 13A-1936C, Vici AG) was switched with a universal valve actuator (EUA02131, Vici AG) to loading position and switched back to injection position after five seconds, and the chromatographic run started.

### Fluidic Connections

Figure S2: Schematic for the fluidic connections interfacing the cooled chip rector.

Connections were made by PTFE tubing (300 µm I.D., 1/16” O.D.) and Idex® Super Flangeless™ nuts and ferrules (Idex) unless otherwise noted. Connections into the threaded glass reactor (female UNF ¼ 28) were terminated with DIBAfit™ gripper ferrules (1/16 steel/PTFE) and nuts (Diba Industries, USA). This was necessary to provide a good sealing. Proper sealing of the connections into the reactor was assured by tightening the fittings, cooling the reactor for 30 min by immersion into a dry ice bed, retightening the fittings to finger tight and slowly warming up the reactor. Tightness was assured by applying pressurized air at 5 bar and closing all exit ports, assuring that pressure was stable.

### Reactant delivery

Reagents were delivered via two syringe pumps (Harvard Apparatus Elite 11, Harvard Apparatus, Holliston, United States) equipped with 1 mL syringes (see syringe preparation for details). One of the two syringes was then filled with the solution of donor and acceptor in anhydrous CH_2_Cl_2_ and the other with activator solution (see Glycosyl donor, acceptor and activator preparation). Quenching solution (1.2 v% pyridine in anhydrous DMF) was delivered via a ML600 syringe pump (5 mL glass/PTFE syringe, Hamilton Bonaduz AG, Bonaduz, Switzerland).

### Glycosyl donor, acceptor and activator preparation

Acceptor and donor solution was prepared as follows: one bead of molecular sieve (3Å, 562 C, Roth) was put into a glass crimp vial (Vial N13-2, CR, c, 13.75x35, flat, Macherey-Nagel) and dried in a vacuum oven (180 °C, ~10 mbar) overnight. The vials were closed immediately with properly fitting rubber septa and cooled. Before opening, the pressure was equalized with anhydrous argon. Donor (0.09 mmol) and acceptor (0.09 mmol) were added into the vial with minimal exposure to air. The vial was crimped with a suitable cap (crimp closure, N 13, alu., center hole, butyl dark gray/PTFE gray, 2.0 mm), and traces of water removed by high vacuum (1*10^-2^ mbar) for 15 min. After filling the vial with argon, 1 mL of anhydrous CH_2_Cl_2_ was added. The so prepared solution was kept in the freezer (−20 °C) for at least three days to allow for scavenging of water traces.

Activator was prepared as follows: A 10 mL glass crimp vial (Vial N20-10, CR, c, 22.5x46, r, DIN, Macherey Nagel) was dried in a vacuum oven (180 °C, ~10 mbar) overnight. After cooling down under inert atmosphere, 140 mg of N-iodosuccinimide were introduced with minimal exposure to air, the vial was crimped with a cap (crimp closure, N 20, alu., center hole, silicone white/PTFE beige, 3.0 mm), and traces of water removed by high vacuum (1*10^-2^ mbar) for 15 min, the vials filled with argon were stored at −20 °C until usage. To create the activator solution (13.4 mM in TfOH, ~12 v% 1,4-dioxane, 0.155 M in NIS), the vial was warmed to rt, the septum heated quickly with a heat gun to remove traces of condensation, and 3.53 mL (4.682 g) of anhydrous CH_2_Cl_2_ introduced, measured by weight. To that, 0.47 mL of a solution of 1 mL of TfOH in 97.9 mL (101.1 g) of 1,4-dioxane (0.1 M) stored in a dried Schlenk flask was added slowly while agitated in a ultrasonication bath. After preparation, the activator solution was immediately used or stored in the freezer for up to three days. To test higher 1,4-dioxane concentration, activator was prepared according to the same protocol, but only 2.75 mL anhydrous CH_2_Cl_2_ (3.648 g) and 0.78 mL of anhydrous 1,4-dioxane were used.

#### Preparation of donor syringe

Donor syringes (Syringe 1001C 1 mL Varian, Hamilton Bonaduz AG, Bonaduz, Switzerland) were dried by heating the syringe body without plunger for 15 min at 120 °C and cooling under an argon stream. The plunger was wetted with one drop of anhydrous CH_2_Cl_2_ and inserted about 1 cm into the syringe body. The syringes were closed on the syringe tip with suitable ¼"-28 fitting and stored until usage. Attention: The plunger tip material is PTFE and rather soft – use caution when taking apart and putting together – wear on the sealing plunger tip can been drastically reduced by only removing the plunger after adding a few drops of ethyl acetate or other suitable solvent from the syringe top. After usage, the syringes should be washed thoroughly.

#### Preparation of activator syringe

Non-metallic syringes (1.0 mL ¼"-28 Tubing Connector and a PE-UHMW-plunger tip seal, article number 2624017, ILS GmbH, Ilmenau, Germany) were connected to high vacuum (10^-2^ mbar) by inserting the syringe tip into vacuum silicone tubing with appropriate internal diameter of Schlenk line with the plunger fully retracted, and left overnight. Pressure was equilibrated with anhydrous argon, the syringe extracted from the tube and immediately closed with ¼”-28 plug. It should be noted that the syringes are not very withstanding to herein used solvents due to the glue used in manufacturing. In our experience reglueing the syringes (2K UHU Plus Endfest, UHU GmbH) gives much better resistance.^[66]^

### Experiment Execution

#### Glycosylation in Flow

####
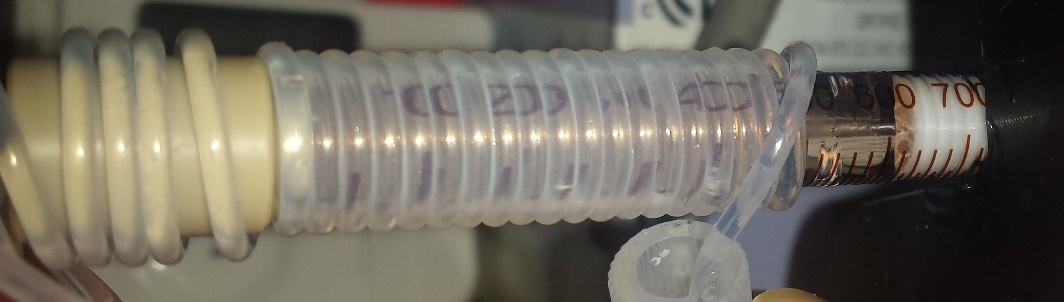


Figure S3: Activator syringe with tubing carrying cooling fluid. Omitted is the employed insulation tube put around the so-cooled syringe.

Glycosyl donor and acceptor were filled into syringe A. Activator was filled into syringe B. Syringe B, after fixed in the syringe pump, was cooled via a custom-made recirculating chiller to ~0 °C, by wrapping ETFE tubing carrying cold ethanol-water mix around the syringe body and applying insulating material from outside (see Figure 3). This prevents a color change in the activator and retains its activating capability for at least 12 h. The syringes were connected to the microreactor inlets one by one. It should be noted that it is important to prime the syringes one by one, this means closing all inlets except for the one for the new syringe connected. The syringe is then inserted into the syringe pump with the outlet pointing up and all air eliminated until only liquid is visible in the tube with the syringe pump. This is done for all syringes successively; the reason is to avoid backflow that can arise in case of compressible volumes, e.g. gas or a not constricted plunger, into the syringe. Before starting a sequence of experiments, the reactor was dried at room temperature, by pumping 2-MeTHF (3 x 2 mL) and methylene chloride 2 x 3 mL at a flowrate of 4 mL/min. This is both to reduce water levels in the reactor and to eliminate gas bubbles caught in the structure of the reactor that would otherwise reduce the internal volume, change the residence time and thereby interfere with acquisition of well-controlled reaction data. Before each individual experiment, the reactor was purged with CH_2_Cl_2_ (1 mL, 4 mL/min) to remove potentially accumulated gas and remaining chemicals of the last reaction. The reactor was set to the according temperature, once this temperature was reached, the pumps were started at a flow rate to accommodate 5 min of residence time: Quencher (0.1 mL/min, 1.2% pyridine in DMF), Activator (5.8 µL/min) and Donor+Acceptor (5.8 µL/min). After 2.5 residence times passed, the sample loop was switched, and a sample injected into LC-ELSD/MS. The reactor effluent was collected for 10 min in a glass vial and reanalyzed by LC-ELSD/MS to make sure the experiment was conducted at steady state and by LC-UVVis/MS. An aliquot of the effluent was evaporated to dryness under reduced pressure, dissolved in CDCl_3_ and submitted to NMR analysis.

#### Glycosylation in Batch

To test the impact of different solvents, 7.2 mg of donor and 1 equiv. of acceptor was introduced into a carefully dried (vacuum oven) crimp vial equipped with a small stir bar and filled with either MeCN or MTBE (0.1 mL). After cooling in an ice bath (ice, NaCl) side by side and at ~−16 °C, activator solution (0.1 mL) was slowly added. After 15 min, the reactions were quenched with anhydrous pyridine in DMF (1.2 v%), the solvent of an aliquot removed under reduced pressure and the sample submitted to NMR-analysis.

Comparison of sampling scale

The donor concentration in the reaction solution was 0.045 M, which was diluted to 5 mM by the quencher stream. Of this, 1 µL, or 5 nmol was used for LC analysis. For IMS analysis, the 5 mM reactor outlet was further diluted to 0.5 mM, and 5 µL or 2.5 nmol were injected into the IMS system. For NMR analysis, at least 100 µL or 0.5 µmol of reactor outlet were used.

## Syntheses

### Synthesis of donor 6 (ethyl 2,3-*O*-dibenzyl-4-azido-6-deoxy-thio-α/β-L-glucopyranoside)

#### Peracetylated L-fucose (S1α):

L-Fucose **S1** (500 mg, 3.05 mmol) and DMAP (11.1 mg, 91.4 µmol) were dissolved in pyridine (10 mL) and the solution was cooled to 0 °C. Ac_2_O (5 mL, precooled to 0 °C) was added and the reaction mixture was further stirred at 0 °C for 2 h. Upon complete conversion (indicated by TLC) the mixture was concentrated under reduced pressure, dissolved in toluene (20 mL) and washed three times with 1 M HCl, H_2_O and brine. The organic layer was dried over Na_2_SO_4_ and concentrate in *vacuo*. The crude product **S1α** was obtained as a colorless syrup (880 mg, 2.65 mmol, 87% yield) and used in the next step without further purification.

**^1^H-NMR** (400 MHz, CDCl_3_): *δ =*6.32 (d, *J*= 2.7 Hz, H-1, 1H), 5.32 (m, H-2, H-3, H-4, 3H), 4.28 – 4.23 (m, H-5, 1H), 2.15 (d, *J* = 12.9 Hz, C(O)CH_3_, 6H), 1.99 (d, *J* = *5.0 Hz*, C(O)CH*_3_*, 3H), 1.14 (d, *J* = 6.5 Hz, CH_3_, 3H) ppm. NMR data were in agreement with those previously reported.^[53,66]^

Spectrum S1: ^1^H-NMR (400 MHz, CDCl_3_) of S1α.

#### Ethyl 2,3,4-O-acetyl-thio-α/β-L-fucopyranoside (S2):

EtSH (241 µL, 3.34 mmol, 1.1 equiv.) was added to a 0.2 M solution of peracetylated intermediate **S1α** (1.01 g, 3.04 mmol) in anhydrous CH_2_Cl_2_ under argon atmosphere. The mixture was cooled to 0 °C and BF_3_∙OEt_2_ (455 µL, 3.95 mmol, 1.3 equiv.) was added dropwise while stirring. The reaction was slowly brought to rt and further stirred for 18 h. Upon complete conversion (indicated by TLC) aqueous saturated NaHCO_3_ solution precooled to 0 °C was added and the aqueous layer was extracted three times with CH_2_Cl_2_. The organic layers were combined, washed with brine, dried over Na_2_SO_4_, concentrated *in vacuo* and purified via column chromatography (hexanes:EtOAc (7:3)). Both anomers were isolated, the α-anomer as colorless oil **S2α** (277 mg, 828 μmol, 27% yield) and the β-anomer **S2β** (525 mg, 1.60 mmol, 53% yield) as colorless crystals.

**S2α: ^1^H-NMR** (400 MHz, CDCl_3_) *δ*= 5.69 (d, *J*= 5.3 Hz, H-1, 1H), 5.29 – 5.19 (m, H-2 H-3 H-4, 3H), 4.51 – 4.46 (m, H-5, 1H), 2.62 – 2.46 (m, SCH_2_CH_3_, 2H), 2.16 (s, (C=O)CH_3_, 3H), 2.07 (s, (C=O)CH_3_, 3H), 1.99 (s, (C=O)CH_3_, 3H), 1.25(t, *J*= 7.4 Hz, SCH_2_CH_3_, 3H), 1.21 (d, *J*= 6.5 Hz, H-6, 3H) ppm.

**S2β:** **^1^H-NMR** (400 MHz, CDCl_3_) *δ*= 5.28 – 5.19 (m, H-2;H-4, 2H), 5.04 (dd, *J =*10.0, 3.4 Hz, H-3, 1H), 4.45 (d, *J =*9.9 Hz, H-1, 1H),3.84 – 3.79 (m, H-5, 1H), 2.79 – 2.65 (m, SC*H_2_*CH_3_, 2H), 2.17 (s, C(O)C*H*_3_, 3H), 2.06 (s, C(O)CH_3_, 3H), 1.98 (s, C(O)CH_3_, 3H), 1.27 (t, *J*= 7.5 Hz, SCH_2_CH*_3_*, 3H), 1.21 (d, *J*= 6.4 Hz, H-6, 3H) ppm. NMR data were in good agreement with those previously reported.^[53]^

Spectrum S2: ^1^H-NMR (400 MHz, CDCl_3_) of S2β.

Spectrum S3: ^1^H ^1^H COSY (CDCl_3_) of S2β.

Spectrum S4: ^1^H-NMR (400 MHz, CDCl_3_) of S2α.

Spectrum S5: ^1^H ^1^H COSY (CDCl_3_) of S2α.

#### Ethyl 2-*O*-benzyl-3,4-*O*-isopropylidene-thio-α-L-fucopyranoside (S3):

Compound **S2α** (1.58 g, 4.73 mmol) was dissolved in MeOH (17 mL). A solution of NaOMe in MeOH (0.5 M, 1.89 mL, 945 µmol, 0.2 equiv.) was added and the mixture stirred at rt overnight. Upon complete conversion (indicated by TLC) the reaction was neutralized with Amberlite IR-120 (H^+^-form), filtered and concentrated under reduced pressure. The product was used without further purification assuming quantitative conversion. The crude was dissolved in anhydrous THF (27 mL) and *p*-toluenesulfonic acid (163 mg, 946 µmol, 0.2 equiv.) and 2,2-dimethoxypropane (1.16 mL, 9.46 mmol, 2.0 equiv.) were added under argon atmosphere. The reaction mixture was stirred at 40 °C for 2 h until complete conversion was indicated by TLC. The reaction was quenched by addition of triethylamine (293 µL, 2.13 mmol, 0.45 equiv.) and H_2_O was added. The aqueous mixture was extracted three times with EtOAc, the combined organic layers washed with brine, dried over MgSO_4_ and concentrated under reduced pressure. The product was obtained as pale yellow crystals (1.14 g, 4.59 mmol, 97% yield).

**^1^H-NMR** (400 MHz, CDCl_3_): δ = 5.29 (d, *J*= 5.0 Hz, H-1, 1H), 4.47 – 4.42 (m, H-5, 1H), 4.06 – 4.04 (m, H-3; H-4, 2H), 4.02- 3.95 (m, H-2, 1H), 2.72 – 2.17 (m, SCH_2_CH_3_, 2H), 2.18 (d, *J*= 6.6 Hz, OH, 1H), 1.53 (s, CH_3_, 3H), 1.35 – 1.29 (m, SCH_2_C*H_3_*; H-6; CH_3_, 9 H) ppm.

**^13^C-NMR** (101 MHz, CDCl_3_): δ = 109.4, (C(CH_3_)), 85.1 (C-1), 76.7, 76.0, 70.3, 64.8 (C-5), 28.2 (CH_3_), 26.2 (S*C*H_2_CH_3_), 25.5 (CH_3_), 16.4 (S*C*H_2_CH_3_/C-6), 15.3 (S*C*H_2_CH_3_/C-6) ppm.

ESI-HRMS *m/z* [M + Na]^+^ calcd for C_11_H_20_NaO_4_S: 271.0980, found 271.0965.

Spectrum S6: ^1^H-NMR (400 MHz, CDCl_3_) of S3.

Spectrum S7: ^13^C-NMR (101 MHz, CDCl_3_) of S3.

Spectrum S8: ^1^H ^13^C HSQC (CDCl_3_) of S3.

#### Ethyl-2-*O*-benzyl-1-thio-α-L-fucopyranoside (S4):

Compound **S3** (1.14 g, 4.59 mmol) was dissolved in anhydrous DMF (37 mL) under argon atmosphere. Subsequently, the reaction mixture was cooled to 0 °C and NaH (734 mg, 60 % dispersion in mineral oil, 18.4 mmol, 4.0 equiv.) was added. The reaction mixture was stirred for 7 min at rt, followed by dropwise addition of BnBr (2.18 mL, 18.4 mmol, 4.0 equiv.) at 0 °C. The reaction mixture was further stirred at 50 °C for 1.5 h. Upon complete conversion (indicated by TLC), the reaction was quenched by addition of ice water. The mixture was extracted three times with a mixture of hexanes/EtOAc (1:1), the combined organic layers were washed with brine, dried over MgSO_4_ and concentrated under reduced pressure. The product was used in the next step without further purification assuming quantitative conversion. A solution of AcOH (94 mL, 80% aqueous) was added to the crude and the mixture was stirred at rt for 18 h. Upon complete conversion (indicated by TLC), the reaction mixture was concentrated under reduced pressure. The crude was further purified by column chromatography (hexanes:EtOAc (1:1)) to give compound **S4** as white solid (907 mg, 3.04 mmol, 66% yield over two steps).

**^1^H-NMR** (CDCl_3_, 400 MHz): δ = 7.40 – 7.29 (m, Ar-H, 5H), 5.50 (d, *J = 5.3 Hz*, H-1, 1H), 4.75 (d, *J =*11.3 Hz, PhCH_2_, 1H), 4.48 (d, *J =*11.3 Hz, PhCH_2_, 1H), 4.31 (m, H-5, 1H), 3.97 (dd, *J =*9.7, 5.3 Hz, H-2, 1H), 3.86 (dd, *J*= 9.7, 3.3 Hz, H-3, 1H), 3.81 – 3.80 (m, H-4, 1H), 2.64 – 2.48 (m, SCH_2_CH_3_; OH, 3H), 1.31 – 1.27 (m, SCH_2_CH_3_; H-6, 6H) ppm.

**^13^C-NMR** (CDCl_3_, 101 MHz): 137.6, 128.7, 128.4, 128.3 (Ar-C), 82.7 (C-1), 75.8 (C-2), 71.8 (PhCH_2_), 71.4 (C-4), 70.5 (C-3), 65.9 (C-5), 24.1 (S*C*H_2_CH_3_), 16.2, 15.0 (SCH_2_*C*H_3_, C-6) ppm.

ESI-HRMS *m/z* [M + Na]^+^ calcd for C_15_H_22_NaO_4_S: 321.1137, found 321.1140.

Spectrum S9: ^1^H-NMR (400 MHz, CDCl_3_) of S4.

Spectrum S10: ^13^C-NMR (101 MHz, CDCl_3_) of S4.

Spectrum S11: ^1^H ^13^C HSQC (CDCl_3_) of S4.

#### Ethyl 2,3-*O*-dibenzyl-thio-α-L-fucopyranoside (S5)

Compound **S4** (889 mg, 2.98 µmol) was dissolved in MeOH (24 mL). Bu_2_SnO (890 mg, 3.58 µmol, 1.2 equiv.) was added and the mixture was stirred for 18 h at 70 °C. Upon complete conversion, the reaction mixture was concentrated under reduced pressure. The residue was dissolved under argon atmosphere in anhydrous DMF (17 mL) and benzyl bromide (425 µL, 3.58 µmol, 1.2 equiv.) was added followed by CsF (588 mg, 3.87 µmol, 1.3 equiv.). The reaction mixture was further stirred for 18 h at rt. Upon complete conversion (indicated by TLC) the reaction mixture was concentrated under reduced pressure, dissolved in CH_2_Cl_2_ (100 mL) and washed with 1 M potassium fluoride (50 mL), water and brine. The organic layer was dried over MgSO_4_ and concentrated under reduced pressure. The crude was further purified via column chromatography (hexanes:EtOAc (7:3)) to give the desired product **S5** as colorless crystals (1.09 g, 2.81 µmol, 95% yield).

**^1^H-NMR** (400 MHz, CDCl_3_): δ = 7.40 – 7.38 (m, ar.H, 2H), 7.37 – 7.28 (m, Ar-H, 8H), 5.43 (d, *J*= 5.6 Hz, H-1, 1H), 4.81 – 4.65 (m, PhCH_2_, 4H), 4.30 – 4.25 (m, H-5, 1H), 4.09 (dd, *J*= 9.7, 5.6 Hz, H-2, 1H), 3.83 – 3.81 (m, H-4, 1H), 3.73 (dd, *J*= 9.7 , 3.4 Hz, H-4, 1H), 2.63 – 2.46 (m, SCH_2_CH_3_, 2H), 2.44 (s, OH, 1H), 1.30 – 1.27 (m, SCH_2_CH_3_; H-6, 6H) ppm.

**^13^C-NMR** (101 MHz, CDCl_3_): δ = 138.3, 138.2, 128.6, 128.5, 128.1, 128.0, 127.94, 127.89 (Ar-C), 78.3 (C-1), 75.3 (C-4), 73.0 (C-2), 72.5 (PhCH_2_), 70.4 (PhCH_2_), 65.8 (C-5), 23.9 (S*C*H_2_CH_3_), 16.3, 15.0 (SCH_2_*C*H_3_, H-6) ppm.

ESI-HRMS *m/z* [M + Na]^+^ calcd for C_22_H_28_NaO_4_S: 411.1606, found 411.1592.

Spectrum S12: ^1^H-NMR (400 MHz, CDCl_3_) of S5.

Spectrum S13: ^13^C-NMR (101 MHz, CDCl_3_) of S5.

Spectrum S14: ^1^H ^13^C HSQC (CDCl_3_) of S5.

#### Ethyl 2,3-*O*-dibenzyl-4-azido-6-deoxy-thio-α-L-glucopyranoside (6)

Compound **S5** (250 mg, 643 μmol) was dissolved in anhydrous CH_2_Cl_2_ (12 mL) under argon atmosphere. Anhydrous pyridine (328 µL, 4.07 mmol, 6.0 equiv.) was added. The solution was cooled down to 0 °C and triflic anhydride (216 µL, 1.29 mmol, 2.0 equiv.) was added. The reaction mixture was further stirred at 0 °C for 1 h. Upon complete conversion (indicated by TLC) the reaction was quenched with ice-cold water, washed three times with ice-cold water and brine. The organic layer was separated and dried over Na_2_SO_4_, filtered and concentrated under reduced pressure to give the triflate intermediate as a yellow solid. The product was readily used for the next step without further purification. The yellow solid was dissolved in anhydrous DMF (12 mL) under argon atmosphere and sodium azide (84 mg, 1.29 mmol, 2.0 equiv.) was added. The reaction mixture was stirred at rt for 18 h. Upon complete conversion (indicated by TLC), the reaction mixture was concentrated under reduced pressure, the residue dissolved in EtOAc and washed with saturated NaHCO_3_ and brine. The organic layer was dried over Na_2_SO_4_, filtered and concentrated under reduced pressure. The crude was further purified by column chromatography (hexanes:EtOAc (9:1)) affording the desired product **6** as colorless solid (198 mg, 478 μmol, 74% yield).

**^1^H-NMR** (400 MHz, CDCl_3_): δ = 7.40 – 7.28 (m, Ar-H, 10H), 5.31 (d, *J*= 5.4 Hz, H-1, 1H), 4.93 (d, *J*= 10.5 Hz, PhCH_2_, 1H), 4.78 (d, *J*= 10.5 Hz, PhCH_2_, 1H), 4.73 (d, *J*= 11.7 Hz, PhCH_2_, 1H), 4.64 (d, *J*= 11.7 Hz, PhCH_2_, 1H), 3.99 – 3.92 (m, H-5, 1H), 3.81 (dd, *J*= 9.4, 5.4 Hz, H-2, 1H), 3.71 (t, *J*= 9.4 Hz, H-3), 3.09 (t, *J*= 9.7 Hz, H-4, 1H), 2.62 – 2.46 (m, SCH_2_CH_3_, 2H), 1.30 – 1.27 (m, SCH_2_CH_3_; H-6, 6H) ppm.

**^13^C-NMR** (101 MHz, CDCl_3_): δ = 138.1, 137.7, 128.6, 128.5, 128.3, 128.1, 128.0 (Ar-C), 83.0 (C-1), 80.4 (C-3), 79.7 (C-2), 75.8 (PhCH_2_), 72.4 (PhCH_2_), 68.0 (C-4), 66.4 (C-5), 24.0 (SCH_2_*C*H_3_), 19.0, 14.9 (SCH_2_*C*H_3_, H-6) ppm.

ESI-HRMS *m/z* [M + Na]^+^ calcd for C_22_H_27_NaN_3_O_5_S: 436.1665, found 446.1630.

Spectrum S15: ^1^H-NMR (400 MHz, CDCl_3_) of 6.

Spectrum S16: ^13^C-NMR (101 MHz, CDCl_3_) of 6.

Spectrum S17: ^1^H ^13^C HSQC (CDCl_3_) of 6.

### Synthesis of analytical reference for disaccharide 2 (2,3-*O*-dibenzyl-4-azido-6-deoxy-β-L-glucopyranosyl-(1→4)2-*O*-methoxy-3,4,6-*O*-tribenzyl-ß-D-glucopyranoside):

All syntheses were performed on a 48.4 μmol (20 mg) scale of glycosyl donor **6**. Different temperatures and solvent mixtures were screened and corresponding yield and ratio of anomers are reported in the following table.

Compound **6** was dissolved in anhydrous solvent mixture (0.5 mL, Table S3) and activated 4 Å molecular sieves (50 mg) were added. Compound **7** (0.8 equiv.) was then added to the mixture. The reaction was cooled to the reported temperature and NIS (1.5 equiv.) was added followed by TfOH (0.16 equiv.). The reaction was stirred at reported temperature for 30 min and subsequently quenched by addition of Et_3_N (100 µL). The mixture was concentrated under reduced pressure and the crude was further purified via column chromatography (hexanes:EtOAc (10:1)). Disaccharide **2** was obtained as a mixture of anomers.

Table S3: Conditions tested for the glycosylation of acceptor 7 glycosyl donor 6. The anomer ratio was determined upon isolation.

| **Entry** | **Temperature [°C]** | **Solvent** | **Yield [%]** | **α% (2)** |
| --- | --- | --- | --- | --- |
| **1** | −20 °C for 30 min | CH_2_Cl_2_ | 80 | 60 |
| **2** | −15 °C for 5 min,  0 °C for 30 min | CH_2_Cl_2_ | 71 | 67 |
| **3** | 0 °C for 30 min | CH_2_Cl_2_ | 46 | 67 |
| **4** | −15 °C for 5 min,  0 °C for 30 min | CH_2_Cl_2_:dioxane  (5:1) | 75 | 67 |
| **5** | 0 °C for 30 min | CH_2_Cl_2_:dioxane  (5:1) | 90 | 83 |

In the analytical data of disaccharide **2**, former glycosyl donor (**6**) is annotated as **a** and former glycosyl acceptor (**7**) is annotated as **b**. Analytical data for **2β:**

**^1^H-NMR** (400 MHz, CDCl_3_): δ*=* 7.35 – 7.24 (m, Ar-H, 25H), 4.98 – 4.90 (m, PhCH_2_, 3H), 4.79 – 4.75 (m, H-1a; PhCH_2_, 4H), 3.69 – 3.65 (m, PhCH_2_, 3H), 4.55 (d, *J*= 12.1 Hz, PhCH_2_, 1H), 4.35 (d, *J*= 7.7 Hz, H-1b, 1H), 3.99 (dd, *J*= 8.0, 6.4 Hz, H-6b, 1H), 3.69 – 3.58 (m, H-3b; H-4b/H-5b;-OCH_3_, 5H), 3.55 – 3.49 (m, H-6b; H-4b/H-5b, 2H), 3.40 – 3.36 (m, H-2b, 1H), 3.33 – 3.27 (m, H-3a; H-2a, 2H), 3.06 – 2.98 (m, H-4a; H-5a, 2H), 1.19 (d, *J*= 5.5 Hz, H-6a, 3H) ppm.

**^13^C-NMR** (101 MHz, CDCl_3_): δ = 138.7, 138.5, 138.3, 137.9, 128.6, 128.5, 128.5, 128.3, 128.25, 128.0, 127.8, 127.8, 127.7, 127.66 (30C, Ar.), 104.6 (C-1b), 102.3 (C-1a), 85.0, 82.8, 82.7, 82.4 (C-2b), 75.7 (PhCH_2_), 75.3 (PhCH_2_), 74.9, 74.4, 73.7 (PhCH_2_), 70.4, 70.2 (C-6b), 68.0, 57.3 (OCH_3_), 18.4 (C-6a) ppm**.**

ESI-HRMS *m/z* [M + Na]^+^ calcd for C_48_H_53_NaN_3_O_9_: 838.3680, found 838.3732.

Analytical data for **2α:**

**^1^H-NMR** (400 MHz, CDCl_3_): δ*=* 7.35 – 7.24 (m, Ar-H, 25H), 5.09 (d, *J*= 3.5 Hz, H-1a, 1H), 5.03 (d, *J*= 10.6 Hz, PhC*H_2_*, 1H), 4.93 (d, *J*= 10.9 Hz, PhC*H_2_*, 1H), 4.84 (d, *J*= 10.5 Hz, PhC*H_2_*, 1H), 4.73 (d, *J*= 10.5 Hz, PhC*H_2_*, 1H), 4.68 – 4.64 (m, PhC*H_2_*, 2H), 4.53 – 4.43 (m, PhC*H_2_*, 3H), 4.32 (d, *J*= 7.7 Hz, H-1b, 1H), 3.87 – 3.82 (m, H-5a; H-6b, 2H), 3.78 – 3.68 (m, H-3a; H-4b/H-5b; H-6b; 3H), 3.62 – 3.57 (m, H-3b; -OCH_3_, 4H), 3.51 – 3.46 (m, H-2b; H4b/H-5b, 2H), 3.43 (dd, *J*= 10.5, 3.5 Hz, H-2a, 1H), 3.01 (t, *J*= 9.8 Hz, H-4a, 1H), 0.93 (d, *J*= 6.1 Hz, H-6a, 3H) ppm.

**^13^C-NMR** (101 MHz, CDCl_3_): δ*=* 138.54, 138.49, 138.47, 138.1, 137.9, 128.61, 128.57, 128.52, 128.48, 128.4, 128.3, 128.27, 128.1, 128.06, 128.04, 128.0, 127.8, 127.77, 127.7, 127.6 (Ar-C), 104.8 (C-1b), 97.3 (C-1a), 82.94, 82.89 (C-3b), 80.6 (C-2a), 80.0, 75.8, 75.7 (PhCH_2_), 75.3, 75.28, 74.8 (PhCH_2_), 74.1 (PhCH_2_),, 73.5 (PhCH_2_), 68.9 (C-6b), 68.6 (C-4a), 66.8 (C-5a), 57.2 (OCH_3_), 18.3 (C-6a) ppm.

ESI-HRMS *m/z* [M + Na]^+^ calcd for C_48_H_53_NaN_3_O_9_: 838.3680, found 838.3729.

Spectrum S18: ^1^H-NMR (400 MHz, CDCl_3_) of 2β.

Spectrum S19: ^13^C-NMR (101 MHz, CDCl_3_) of 2β.

Spectrum S20: ^1^H ^13^C HSQC (CDCl_3_) of 2β.

Spectrum S21: ^1^H-NMR (400 MHz, CDCl_3_) of 2α.

Spectrum S22: ^13^C-NMR (101 MHz, CDCl_3_) of 2α.

Spectrum S23: ^1^H ^13^C HSQC (CDCl_3_) of 2α.

### Synthesis of analytical reference for compound 3

All syntheses were performed on a 0.11 mmol (60 mg) scale of glycosyl donor **8**. α:β-anomer ratio of **3** was determined by ^1^H-NMR. The reaction conditions and corresponding yield and ratio of anomers are reported in Table 2.

*N*-(Benzyl)-benzyloxycarbonyl-5-aminopentan-1-ol (**9**) (1 equiv.) and 4-methylphenyl 2,3,4-tri-*O*-benzyl-1-thio-β-L-fucopyranoside donor **8** (60 mg, 0.11 mmol) were dissolved in anhydrous CH_2_Cl_2_ (1 mL) and stirred with molecular sieves (4 Å, 200 mg) at rt under Ar atmosphere. After 1 h, the reaction mixture was cooled to the target temperature and NIS (1.2 equiv.) was added to the solution, followed by dropwise addition of TfOH (0.2 equiv.). The solution was stirred for 25 min, the cooling bath removed subsequently and the the reaction warmed to rt. Et_3_N (0.2 equiv.) was added to the solution and the mixture was stirred at rt for 30 min. The reaction was diluted with CH_2_Cl_2_ and washed with saturated aqueous Na_2_S_2_O_3_ solution, and with saturated aqueous NaHCO_3_ solution. The organic phase was dried with Na_2_SO_4_, filtered, conentrated under reduced pressure and subsequently purified by column chromatography (hexane:EtOAc = 5:1🡪2:1). The product-containing fractions were combined, and dried *in vacuo*. The anomer ratio was determined by integration of H-1 signals in ^1^H-NMR.

Table S4: Stereoselectivity of glycosylation 3 of linker 9 with donor 8 under different reaction conditions.

| **Entry** | **Temperature [°C]** | **Yield [%]** | **α[%] (3)** |
| --- | --- | --- | --- |
| **1** | −10 to 0 °C for 30 min | 88 | 60 |
| **2** | −40 to 0 °C for 30 min | 83 | 20 |

Analytical data for **3α:**

**^1^H-NMR** (400 MHz, CDCl_3_) δ 7.42 – 7.12 (m, 25H), 5.17 (d, *J* = 10.9 Hz, 2H), 4.97 (d, *J* = 11.6 Hz, 1H), 4.86 (d, *J* = 11.7 Hz, 1H), 4.80 (d, *J* = 12.1 Hz, 1H), 4.73 (d, *J* = 11.8 Hz, 2H), 4.65 (d, *J* = 11.9 Hz, 2H), 4.48 (d, *J* = 8.2 Hz, 2H), 4.01 (dd, *J* = 10.1, 3.7 Hz, 1H), 3.94 – 3.87 (m, 1H), 3.86 – 3.77 (m, 1H), 3.64 (s, 1H), 3.58 – 3.30 (m, 2H), 3.28 – 3.14 (m, 2H), 1.66 – 1.46 (m, 3H), 1.34 – 1.19 (m, 3H), 1.08 (d, *J* = 6.5 Hz, 3H).

**^13^C-NMR** (101 MHz, CDCl_3_) δ 128.55, 128.48, 128.37, 128.30, 128.20, 127.94, 127.83, 127.60, 127.45, 97.52, 79.47, 77.72, 77.35, 77.24, 77.04, 76.72, 76.50, 74.81, 73.29, 67.97, 67.17, 66.14, 29.72, 29.21, 16.67, 14.15.

ESI-HRMS *m/z* [M + H]^+^ calcd for C_47_H_54_NO_7_: 744.3900, found 744.3846.

Analytical data for **3β**:

**^1^H-NMR** (400 MHz, CDCl_3_) δ 7.42 – 7.10 (m, 25H), 5.16 (d, *J* = 9.8 Hz, 2H), 4.98 (d, *J* = 11.8 Hz, 1H), 4.93 – 4.86 (m, 1H), 4.82 – 4.67 (m, 4H), 4.50 – 4.44 (m, 2H), 4.30 – 4.23 (m, 1H), 3.92 – 3.82 (m, 1H), 3.82 – 3.74 (m, 1H), 3.55 (d, *J* = 2.9 Hz, 1H), 3.50 (dd, *J* = 9.7, 2.9 Hz, 1H), 3.46 – 3.36 (m, 2H), 4.27 – 3.12 (m, 2H), 1.65 – 1.45 (m, 3H), 1.38 – 1.21 (m, 3H), 1.17 (d, *J* = 6.3 Hz, 3H).

**^13^C-NMR** (101 MHz, CDCl_3_) δ 128.56, 128.53, 128.46, 128.38, 128.27, 128.13, 127.84, 127.57, 127.54, 127.49, 127.23, 103.82, 82.55, 79.47, 77.35, 77.24, 77.04, 76.72, 76.26, 75.08, 74.53, 73.18, 70.27, 67.14, 50.47, 47.23, 29.73, 29.47, 22.72, 16.90.

ESI-HRMS *m/z* [M + H]^+^ calcd for C_47_H_54_NO_7_: 744.3900, found 744.3914.

Spectrum S24: ^1^H-NMR (400 MHz, CDCl_3_) of 3α.

Spectrum S25: ^13^C-NMR (101 MHz, CDCl_3_) of 3α.

Spectrum S26: ^1^H ^13^C HSQC (CDCl_3_) of 3α.

Spectrum S27: ^1^H-NMR (400 MHz, CDCl_3_) of 3β.

Spectrum S28: ^13^C-NMR (101 MHz, CDCl_3_) of 3β.

Spectrum S29: ^1^H ^13^C HSQC (CDCl_3_) of 3β.

### Synthesis of analytical reference of disaccharide 1 (2,3,6-O-Tribenzyl-4-O-Fluorenylmethoxycarbonyl-D-glucopyranosyl-(1→6)-O-1-methoxy-2,3,4-O-tribenzyl-a-D-mannopyranoside)

Thioglycoside **4** (30.0 mg, 4.2 mmol) and 6-OH-mannoside **5** (19.4 mg, 4.2 mmol) were dissolved in anhydrous CH_2_Cl_2_ (450 µL) under argon in a 2 mL crimp vial dried at 180 °C under reduced pressure over night. One large molecular sieve bead (~3 mm, 3Å) was added. The solution was stirred for 1 h at rt and subsequently cooled to −15 °C in an ice bath (ice, NaCl). Activator solution (0.45 mL, 14 µM TfOH, 0.15 M NIS, see “Glycosyl donor, acceptor and activator preparation” for details) was added dropwise via syringe. Complete conversion (indicated by TLC, Hexanes w 20% EtOAc, Rf acceptor 0.09, Rf donor 0.53, Rf product 0.25) was obtained after 5 min, upon which the mixture was quenched with pyridine in CH_2_Cl_2_ (4.5 mL, 1.2 v%), washed with aqueous solution of ascorbic acid, three times water and brine. The organic layer was separated, dried over Na_2_SO_4_ and concentrated *in* *vacuo*. The crude product was obtained as a colorless syrup (40 mg) and anomers separated via column chromatography (toluene:EtOAc 95:5). Both anomers **1α** and **1β** were obtained as clear oil.

Analytical data for **1α**:

**R_f_** (8% EtOAc in toluene): 0.25.

**^1^H-NMR** (400 MHz, CDCl_3_): *δ* = 7.76 (d, J = 7.5 Hz, 2H), 7.56 (dd, J = 7.6, 1.0 Hz, 1H), 7.53 (dd, J = 7.5, 1.0 Hz, 1H), 7.43 – 7.09 (m, 34H), 5.12 (d, J = 3.4 Hz, 1H), 4.96 (dd, J = 10.2, 9.3 Hz, 1H), 4.91 (d, J = 11.1 Hz, 1H), 4.85 (d, J = 11.4 Hz, 1H), 4.72 (d, J = 1.8 Hz, 1H), 4.69 – 4.56 (m, 8H), 4.56 – 4.42 (m, 2H), 4.34 – 4.20 (m, 2H), 4.11 (t, J = 7.3 Hz, 1H), 4.07 – 3.96 (m, 3H), 3.94 – 3.84 (m, 3H), 3.84 – 3.74 (m, 2H), 3.65 – 3.55 (m, 2H), 3.51 (dd, J = 10.8, 4.3 Hz, 1H), 3.29 (s, 3H).

**DEPT135** (101 MHz, CDCl_3_) δ 128.31, 128.27, 128.20, 128.16, 128.14, 128.07, 127.89, 127.79, 127.74, 127.70, 127.57, 127.50, 127.33, 127.09, 127.08, 125.11, 125.05, 119.96, 98.84, 96.35, 82.11, 80.21, 79.47, 78.59, 75.03, 74.88, 74.79, 74.72, 73.45, 72.78, 72.61, 72.08, 71.63, 69.82, 68.64, 68.20, 65.97, 54.67, 46.61.

HRMS (ESI/Q-TOF) m/z: [M + Na]^+^ Calcd for C_70_H_70_O_13_Na 1141.4708; Found 1141.4802.

Analytical data for **1β**:

**R_f_** (8% EtOAc in toluene): 0.18.

**^1^H-NMR** (400 MHz, CDCl_3_) δ 7.76 (d, J = 7.5 Hz, 2H), 7.57 (d, J = 8.0 Hz, 1H), 7.51 (d, J = 7.4 Hz, 1H), 7.27 (m, 34H), 5.02 (d, J = 10.9 Hz, 1H), 4.90 – 4.80 (m, 2H), 4.78 (s, 1H), 4.71 (s, 2H), 4.66 (dd, J = 11.0, 3.7 Hz, 2H), 4.59 (s, 2H), 4.52 (s, 2H), 4.50 (d, J = 11.1 Hz, 1H), 4.44 (d, J = 7.7 Hz, 1H), 4.33 – 4.20 (m, 3H), 4.10 (t, J = 7.3 Hz, 1H), 3.99 – 3.87 (m, 2H), 3.84 – 3.74 (m, 4H), 3.70 – 3.59 (m, 4H), 3.54 (dd, J = 9.3, 7.7 Hz, 1H), 3.26 (s, 3H).

**DEPT135** (101 MHz, CDCl_3_) δ 128.27, 128.24, 128.19, 128.15, 127.82, 127.80, 127.73, 127.67, 127.58, 127.50, 127.46, 127.12, 127.08, 125.10, 125.00, 119.96, 103.89, 98.86, 81.64, 81.59, 80.12, 75.48, 75.34, 74.95, 74.83, 74.80, 74.37, 73.52, 72.80, 72.68, 71.90, 71.21, 69.96, 69.66, 69.10, 55.27, 54.73, 46.57.

Spectrum S30: ^1^H-NMR (400 MHz, CDCl_3_) of 1α.

Spectrum S31: DEPT135 NMR (101 MHz, CDCl_3_) of 1α.

Spectrum S32: ^1^H ^13^C HSQC (CDCl_3_) of 1α.

Spectrum S33: ^1^H-NMR (400 MHz, CDCl_3_) of 1β (400 MHz, CDCl_3_).

Spectrum S34: DEPT135 (101 MHz, CDCl_3_) of 1β.

Spectrum S35: ^1^H ^13^C HSQC (CDCl_3_) of 1β.

|  |
| --- |
|  |

## Raw data of Analysis of Glycosylations

### Model Glycosylation 1 at different temperatures

**

Figure S4: Model Glycosylation 1.

Spectrum S36: IMS-spectra of glycosylation 1 reaction crude (linked to Fig. 2 in the main text).

Spectrum S37: ^1^H-NMR (400 MHz, CDCl_3_) of glycosylation 1 crude in flow at 20 °C with the signal at 3.26 ppm for 1βand 3.29 ppm for 1α (entry 1 in Figure S36).

Spectrum S38: ^1^H-NMR spectrum (400 MHz, CDCl_3_) of glycosylation 1 crude in flow at 0 °C with the signal at 3.26 ppm for 1β and 3.29 ppm for 1α (entry 2 in Figure S36).

Spectrum S39: ^1^H-NMR spectrum (400 MHz, CDCl_3_) of glycosylation 1 crude in flow at −15 °C with the signal at 3.26 ppm for 1β and 3.29 ppm for 1α (entry 3 in Figure S36).

Spectrum S40: ^1^H-NMR spectrum (400 MHz, CDCl_3_) of glycosylation 1 crude in flow at −30 °C with the signal at 3.26 ppm for 1β and 3.29 ppm for 1α (entry 4 in Figure S36).

Spectrum 41: ^1^H-NMR spectrum (400 MHz, CDCl_3_) of glycosylation 1 crude in flow at −35 °C with the signal at 3.26 ppm for 1β and 3.29 ppm for 1α (entry 5 in Figure S36).

Spectrum S42: ^1^H-NMR spectrum (400 MHz, CDCl_3_) of glycosylation 1 crude in flow at −55 °C with the signal at 3.26 ppm for 1βand 3.29 ppm for 1α (entry 6 in Figure S36).

### Model Glycosylation 1 with different solvent additives

**

Figure S5: Model Glycosylation 1 with solvent additives in batch.

Spectrum S43: ^1^H-NMR spectrum (400 MHz, CDCl_3_) of glycosylation 1 crude in batch at −18 °C with the signal at 3.26 ppm for 1βand 3.29 ppm for 1α (entry 8 in Figure 44).

Spectrum S44: ^1^H-NMR spectrum (400 MHz, CDCl_3_) of glycosylation 1 crude in batch at −18 °C in MTBE/CH_2_Cl_2_ solvent mix with the signal at 3.26 ppm for 1βand 3.29 ppm for 1α (entry 9 in Figure S3).

Spectrum S45: ^1^H-NMR spectrum (400 MHz, CDCl_3_) of glycosylation 1 crude in batch at −18 °C in MeCN/CH_2_Cl_2_ solvent mix with the signal at 3.26 ppm for 1β and 3.29 ppm for 1α (entry 10 in Figure S3).

### Model glycosylation 2 at different temperatures

Figure S6: Glycosylation 2 at different 1,4-dioxane concentrations and temperatures.

Spectrum S46: ^1^H-NMR spectrum (400 MHz, CDCl_3_) of glycosylation 2 crude in flow at 0 °C in with 6 v% 1,4-dioxane with the deconvoluted doublet at 4.32 ppm for 2α and the deconvoluted doublet at 4.35 ppm for 2β, the doublet at 4.33 ppm corresponds to unreacted acceptor 7 (entry 1 in Figure S 4).

Spectrum S47: ^1^H-NMR spectrum (400 MHz, CDCl_3_) of glycosylation 2 crude in flow at −20 °C in with 6 v% 1,4-dioxane with the deconvoluted doublet at 4.32 ppm for 2α and the deconvoluted doublet at 4.35 ppm for 2β, the doublet at 4.33 ppm corresponds to unreacted acceptor 7 (entry 2 in Figure S 4).

Spectrum S48: ^1^H-NMR spectrum (400 MHz, CDCl_3_) of glycosylation 2 crude in flow at −40 °C in with 6 v% 1,4-dioxane with the deconvoluted doublet at 4.32 ppm for 2α and the deconvoluted doublet at 4.35 ppm for 2β, the doublet at 4.33 ppm corresponds to unreacted acceptor 7 (entry 3 in Figure S 4).

Spectrum S49: ^1^H-NMR spectrum (400 MHz, CDCl_3_) of glycosylation 2 crude in flow at 0 °C in with 17 v% 1,4-dioxane with the deconvoluted doublet at 4.32 ppm for 2α and the deconvoluted doublet at 4.35 ppm for 2β, the doublet at 4.33 ppm corresponds to unreacted acceptor 7 (entry 4 in Figure S 4).

Spectrum S50: ^1^H-NMR spectrum (400 MHz, CDCl_3_) of glycosylation 2 crude in flow at −20 °C in with 17 v% 1,4-dioxane with the deconvoluted doublet at 4.32 ppm for 2α and the deconvoluted doublet at 4.35 ppm for 2β, the doublet at 4.33 ppm corresponds to unreacted acceptor 7 (entry 5 in Figure S4).

Spectrum S51: ^1^H-NMR spectrum (400 MHz, CDCl_3_) of glycosylation 2 crude in flow at −40 °C in with 17 v% 1,4-dioxane with the deconvoluted doublet at 4.32 ppm for 2α and the deconvoluted doublet at 4.35 ppm for 2β, the doublet at 4.33 ppm corresponds to unreacted acceptor 7 (entry 6 in Figure S4).

| Spectrum S52: IMS-spectra for glycosylation 2 reaction crude (linked to Fig. 3 in the paper).   |
| --- |
|  |

Figure S7: Online LC-ELSD Chromatograms of Glycosylation 2.

Figure S8: Offline LC-ELSD Chromatograms of Glycosylation 2 purified anomers containing the same external contaminants at time < 10 min..

Figure S9: Offline LC-ELSD Chromatograms of Glycosylation 2.

Figure S10: Pure reference compounds of glycosylation 2 in offline LC-ELSD

Figure S11: Offline LC-UVVis chromatograms of Glycosylation 2.

Figure S12: Reference compounds of Glycosylation 2 in LC-UVVis.

### Model Glycosylation 3 (crude reaction mixture ^1^H NMR)

Figure 13: Glycosylation of amino alcohol 9 with fucose donor 8 to produce glycosides 3α and 3β.

Spectrum S53: Reaction crude of glycosylation 3 with signal at 4.28 ppm for 3β and signal at 4-4.04 ppm for 3α..

# References

[52] D. B. G. Williams, M. Lawton, *J. Org. Chem.* **2010**, *75*, 8351–8354.

[53] M. Calosso, D. Charpentier, M. Vaillancourt, M. Bencheqroun, G. St-Pierre, B. C. Wilkes, Y. Guindon, *ACS Med. Chem. Lett.* **2012**, *3*, 1045–1049.

[54] S. Arafuka, N. Koshiba, D. Takahashi, K. Toshima, *Chem. Commun.* **2014**, *50*, 9831–9834.

[55] G. Fittolani, Non-Natural Oligosaccharides: From Structural Studies to the Design of Synthetic Carbohydrate Materials, PhD Thesis, Freie Universität, **2022**.

[56] T. Tyrikos-Ergas, E. T. Sletten, J.-Y. Huang, P. H. Seeberger, M. Delbianco, *Chem. Sci.* **2022**, *13*, 2115–2120.

[57] S. Zhang, P. H. Seeberger, *Chemistry A European J* **2021**, *27*, 17444–17451.

[58] C. Wang, Q. Li, H. Wang, L.-H. Zhang, X.-S. Ye, *Tetrahedron* **2006**, *62*, 11657–11662.

[59] R. Castelli, H. S. Overkleeft, G. A. Van Der Marel, J. D. C. Codée, *Org. Lett.* **2013**, *15*, 2270–2273.

[60] V. Belakhov, E. Dovgolevsky, E. Rabkin, S. Shulami, Y. Shoham, T. Baasov, *Carbohydrate Research* **2004**, *339*, 385–392.

[61] Y. Zhu, M. Delbianco, P. H. Seeberger, *J. Am. Chem. Soc.* **2021**, *143*, 9758–9768.

[62] C. Thoben, C.-R. Raddatz, M. Lippmann, Z. Salehimoghaddam, S. Zimmermann, *Talanta* **2021**, *233*, 122579.

[63] J. Boschmans, S. Jacobs, J. P. Williams, M. Palmer, K. Richardson, K. Giles, C. Lapthorn, W. A. Herrebout, F. Lemière, F. Sobott, *Analyst* **2016**, *141*, 4044–4054.

[64] A. L. Heaton, P. B. Armentrout, *J. Phys. Chem. A* **2008**, *112*, 10156–10167.

[65] S. Lee, T. Wyttenbach, M. T. Bowers, *International Journal of Mass Spectrometry and Ion Processes* **1997**, *167–168*, 605–614.

[66] D. Angelone, A. J. S. Hammer, S. Rohrbach, S. Krambeck, J. M. Granda, J. Wolf, S. Zalesskiy, G. Chisholm, L. Cronin, *Nat. Chem.* **2021**, *13*, 63–69.

[67] B. Ruttens, P. Kováč, *Synthesis* **2004**, *2004*, 2505–2508.
